# SARS-CoV-2 Viral Shedding and Transmission Dynamics: Implications of WHO COVID-19 Discharge Guidelines

**DOI:** 10.3389/fmed.2021.648660

**Published:** 2021-06-17

**Authors:** Kingsley Badu, Kolapo Oyebola, Julien Z. B. Zahouli, Adeniyi Francis Fagbamigbe, Dziedzom K. de Souza, Natisha Dukhi, Ebenezer F. Amankwaa, Mai F. Tolba, Augustina A. Sylverken, Lydia Mosi, Priscilla Kolibea Mante, Damaris Matoke-Muhia, Nowsheen Goonoo

**Affiliations:** ^1^African Academy of Sciences Affiliates, Nairobi, Kenya; ^2^Department of Theoretical and Applied Biology, Kwame Nkrumah University of Science and Technology, Kumasi, Ghana; ^3^Biochemistry and Nutrition Department, Nigerian Institute of Medical Research, Lagos, Nigeria; ^4^Department of Zoology, Faculty of Science, University of Lagos, Lagos, Nigeria; ^5^Centre Suisse de Recherches Scientifiques en Côte d'Ivoire, Abidjan, Côte d'Ivoire; ^6^Centre d'Entomologie Médicale et Vétérinaire, Université Alassane Ouattara, Bouaké, Côte d'Ivoire; ^7^Department of Epidemiology and Medical Statistics, Faculty of Public Health, College of Medicine, University of Ibadan, Ibadan, Nigeria; ^8^Division of Population and Behavioral Sciences, School of Medicine, St. Andrews University, St. Andrews, United Kingdom; ^9^College of Health Sciences, Noguchi Memorial Institute for Medical Research, University of Ghana, Accra, Ghana; ^10^Human and Social Capabilities Division, Human Sciences Research Council, Cape Town, South Africa; ^11^Department of Geography and Resource Development, University of Ghana, Accra, Ghana; ^12^Department of Pharmacology and Toxicology, Faculty of Pharmacy, Ain Shams University, Cairo, Egypt; ^13^The Center of Drug Discovery Research and Development, Faculty of Pharmacy, Ain Shams University, Cairo, Egypt; ^14^School of Life and Medical Sciences, University of Hertfordshire Hosted by Global Academic Foundation, New Administrative Capital, Egypt; ^15^Kumasi Centre for Collaborative Research in Tropical Medicine, Kwame Nkrumah University of Science and Technology, Kumasi, Ghana; ^16^West African Centre for Cell Biology of Infectious Diseases, University of Ghana, Accra, Ghana; ^17^Department of Biochemistry, Cell and Molecular Biology, University of Ghana, Accra, Ghana; ^18^Department of Pharmacology, Kwame Nkrumah University of Science and Technology, Kumasi, Ghana; ^19^Centre for Biotechnology Research and Development, Kenya Medical Research Institute, Nairobi, Kenya; ^20^Biomaterials, Drug Delivery and Nanotechnology Unit, Center for Biomedical and Biomaterials Research (CBBR), University of Mauritius, Reduit, Mauritius

**Keywords:** COVID19, viral shedding, discharge recommendations, transmission dynamics, SARS-CoV-2

## Abstract

The evolving nature of the severe acute respiratory syndrome coronavirus 2 (SARS-CoV-2) has necessitated periodic revisions of COVID-19 patient treatment and discharge guidelines. Since the identification of the first COVID-19 cases in November 2019, the World Health Organization (WHO) has played a crucial role in tackling the country-level pandemic preparedness and patient management protocols. Among others, the WHO provided a guideline on the clinical management of COVID-19 patients according to which patients can be released from isolation centers on the 10th day following clinical symptom manifestation, with a minimum of 72 additional hours following the resolution of symptoms. However, emerging direct evidence indicating the possibility of viral shedding 14 days after the onset of symptoms called for evaluation of the current WHO discharge recommendations. In this review article, we carried out comprehensive literature analysis of viral shedding with specific focus on the duration of viral shedding and infectivity in asymptomatic and symptomatic (mild, moderate, and severe forms) COVID-19 patients. Our literature search indicates that even though, there are specific instances where the current protocols may not be applicable ( such as in immune-compromised patients there is no strong evidence to contradict the current WHO discharge criteria.

## Background

On the 27th of May 2020, the World Health Organization (WHO) provided an update to guide the clinical management of COVID-19, wherein criteria for discharging patients were recommended (1). As part of the clinical control of COVID-19, a COVID-19 infected person can be allowed to go home (regardless of location or disease severity), 10 days following clinical manifestations, with at least 72 additional hours whereby no symptoms have been noted (without fever and without respiratory symptoms). This is in contrast with the WHO's earlier (12 January 2020) recommendations which required the patient to be clinically recovered and to have two negative reverse transcriptase quantitative polymerase chain reaction (RT-qPCR) results on consecutive specimens taken at least 1 day apart (2).

Even though the WHO affirms that its recommendations are based on evidence that patients without symptoms may continue to shed the virus for several weeks, the viral particles are not infectious and are of no threat for subsequent transmission. The main reasons that necessitated the May 3rd, 2020 discharge guidelines included apparent challenges and lack of resources in areas of intense transmission (1). Indeed, in some regions with high SARS-CoV-2 transmission rates, implementation of the earlier discharge protocols had been challenging (3). This is largely due to limited laboratory supplies, equipment and personnel, especially outside hospital settings. Furthermore, long isolation periods for patients with positive PCR results but without any symptoms were found to negatively affect their physical and mental well-being (4). In several countries, this was further compounded by insufficient contact tracing and test material/labor to meet the first WHO discharge recommendations (3).

Given the potential strengths and weaknesses of the May 2020 guidelines, it is important to interrogate the updated recommendations and criteria based on scientific evidence and context-specific operational challenges. In particular, it is important to reflect and provide answers to these questions: (1) Are RT-PCR tests reliable enough? (2) From which site do we take specimens? (3) Is there a correlation between viral load and disease severity & patient status (symptomatic and asymptomatic)? (4) Does the duration of viral shedding correlate with infectivity? Empirical evidence on the above questions will help further guide and improve discharge protocols that can shorten patient isolation and minimize transmission risks of convalescing patients. This review critically analyses existing literature on SARS-CoV-2 viral shedding dynamics and infectivity in convalescing patients.

## Methods

Authors attempted to assess in-depth a multitude of COVID-19 reports in PubMed, Google Scholar and Scopus. The following keywords were used as search terms: viral shedding, COVID-19, SARS-CoV-2, transmission, infectivity, viral load, dynamics, viral viability, and WHO discharge guidelines. The date of last search was April 2021. Concerned about the delays between completion of studies and their publication and taking into consideration the high turnover of publications on COVID-19 pandemic, manuscripts on preprint servers were also considered (Research Square medRxiv dimensions and BioRxiv). Articles identified by the searches were reviewed along with any relevant references cited within them. Articles were excluded if the full texts were not available or if they were not published in English.

## Dynamics of SARS-CoV-2

Understanding the dynamics and duration of infectivity of SARS-CoV-2 is critical in the setting up of protocols guiding public health for quarantine, isolation and contact tracing. The period of infectivity is the space of time within which a naive individual may acquire the infection from an infected person (5). The clinical spectrum of SARS-CoV-2 is very heterogeneous and the viral RNA shedding patterns vary in terms of shedding duration and viral load depending on the nature (e.g., age, sex) and status (e.g., asymptomatic, pre-symptomatic, symptomatic) of patients, severity of disease (e.g., mild and severe COVID-19), and type of sample tested (e.g., samples of upper respiratory tract and lower respiratory tract).

### Viral Loads in Different Specimens

Viral loads are generally quantified via RT-qPCR. In most studies, viral loads from upper respiratory tract samples peaked at the time of symptom onset and lasted for a few days, but gradually decreased over the following 1–3 weeks (6–8). Viral loads from stool samples peaked 3–4 weeks after symptom onset and followed an erratic pattern (6). Inconsistency in reported data was noted in terms of viral load dynamics for upper respiratory samples depending on the location where the specimen was taken (9, 10). Indeed, few reports concluded on higher viral RNA in nasal swabs (9) compared to others who noted higher viral loads in throat specimens (10). However, the conclusion from the study conducted by Zou et al. (9) needs cross-verification since the sample size was only 18 patients. The amount of viral RNA found in the upper respiratory tract, lower respiratory tract (sputum) and fecal samples followed different trends. Maximum viral load was noted in upper respiratory tract (URT) samples within 1 week of symptom onset and decreased rather consistently becoming practically undetectable 2 weeks after symptom onset (7, 8). On the other hand, maximum viral load in lower respiratory tract (sputum) samples were noted later, on average 2 weeks after symptom onset (11, 12) and displayed higher viral loads than upper respiratory tract samples (10, 13). Lower respiratory tract (LRT) samples have shown increased sensitivity compared to URT specimens (14–16). Indeed, compared to URT (nasal and throat), LRT samples resulted in the highest positive rate for all stages of COVID-19 infection (14). However, the use of LRT specimens may be limited by the fact that only a small portion of COVID-19 (28%) patients produce sputum (17). Moreover, even though viral RNA could not be detected in URT samples, LRT [bronchoalveolar lavage fluid (BALF)] tested positive (14). Based on these studies, detection of viral load in LRT samples may be a good strategy to follow COVID-19 evolution particularly in patients displaying low viral loads. However, we note the technical challenges associated with collection of sputum or BALF, especially in patients who are not intubated.

### Variation of Viral Loads and Infectivity With Disease Severity or Presence/Absence of Symptoms

Viral load was directly linked with disease severity with higher viral loads leading to severe forms of the infection (6, 18). Indeed, in line with Zheng et al. (6), Liu et al. (18) found that the average viral load in severe cases (*n* = 76) was higher than in mild cases. Consequently, patients with severe COVID-19 infection had significantly higher viral loads in respiratory samples compared to those presenting with mild symptoms. In addition, Zheng et al. (6), noted no significant difference in viral loads in stool and serum samples between patients with mild and severe disease (6). Mild cases correlated with early viral clearance while patients with severe symptoms continued shedding the virus for a longer period of time (6, 18). Indeed, RT-PCR results were repeatedly negative for 90% of these patients by day 10 post-symptom-onset in comparison to severe cases, whereby RT-PCR results were positive at or beyond day 10 post-onset (18). Thus, these studies suggest that the initial viral load impacts on the length of hospital stay. However, both studies display limitations such as insufficient sample size. In addition, the viral load is dependent on the quality of collected samples and hence the viral load level reported in the studies only partly reflects the amount of virus present in the body. And finally, viral shedding was assessed through PCR tests and the latter cannot distinguish between viable and non-viable virus and does not reflect the replication level of the virus in different tissues. Taken altogether, there is no clear evidence to conclude on the relationship between viral load and infectivity.

With respect of viral load and disease severity, there is evidence supporting that higher viral load leads to severe cases. The viral load of sputum specimens collected from the lower respiratory tract and tested at baseline was closely linked to disease severity (19). In particular, patients displaying a higher baseline viral load were more likely to develop a severe form of COVID-19. This important observation suggests that the early administration of antiviral treatment can reduce the risk of disease progression and mortality.

Some studies report little or no difference in viral loads between pre-symptomatic, and those displaying and not displaying symptoms (20, 21). For instance, Arons et al. (22) found almost similar viral loads between asymptomatic and pre-symptomatic patients with the median cycle threshold (Ct) values of 25.5 and 23.1, respectively, (lower Ct values infer higher viral loads) (22) and therefore the use of symptom based strategies is insufficient in the prevention of SARS-CoV-2 transmission. On the other hand, average Ct values for asymptomatic patients was significantly higher compared to those in the early stages of infection (23). Both symptomatic and asymptomatic groups displayed the same time frame of viral shedding (7 vs. 8 days) which suggests that patients without COVID-19 symptoms can still transmit the virus. Moreover, two studies confirmed the peak of transmissibility near, and even before symptom onset (24, 25). Even though a reduction in infectivity was noted in symptomatic patients 7–10 days after onset of symptoms (8), viable virus could still be isolated from upper and lower respiratory samples 13 and 18 days, respectively, after symptom onset (18, 22).

SARS-CoV-2 infection is primarily diagnosed based on detecting the presence of viral RNA by molecular testing, usually by RT-PCR in a specimen produced by the patient. However, detection of viral RNA does not necessarily mean that a person is infectious (26). Determination of the presence of viable virus may be accomplished by monitoring the ability of SARS-CoV-2 to replicate in laboratory-based cell culture. It is however important to know whether the infected case has mild-to-moderate symptoms or are patients with severe-to-critical illnesses, or immunocompromised. Some studies have provided evidence to suggest that COVID-19 patients with mild- to moderate illness are highly unlikely to be infectious beyond 10 days from symptom onset (19, 27). On the contrary, current evidence also indicates that patients with severe-to-critical illness, and those who are immunocompromised, may be infectious for a prolonged period, possibly for 20 days or more (28–30). All these studies point to the fact that one should consider disease severity, viral loads as well as immunosuppression status of the patients involved before determining whether a case should be quarantined according to WHO standards.

### Dynamics of Transmission

At the early stage of COVID-19 infection, an innate immune response characterized by interferon and cytokines is triggered. This slows down the replication and spread of the virus, until the adaptive immune response (humoral and cellular) starts to clear the infection. Variation in the time of activation of the adaptive immune response in different individuals implies that the SARS-CoV-2 virus can continue replicating and remains viable (31). In a recent study, it was found that interferons play a key role in suppressing the virus in the upper airway, while macrophages are primarily responsible for viral clearance in the lungs, mononuclear phagocyte system (MPS), and other systemic sites (32). Therefore, the rate at which lung and MPS macrophages clear the virus influences the viral load in both the lungs and the plasma (32). In patients with defective immune systems, viral clearance will depend on the excretion of the virus through the bile duct, which can take weeks.

In a case series conducted by Vetter et al. (33), the authors monitored the shedding of viral SARS-CoV-2 RNA and characterized the immune response kinetics. Strong innate responses was noted characterized by an increase in type I interferon and proinflammatory cytokines, as well as a significant increase in intermediate monocytes with activation, differentiation, and migration patterns as from 2 days following symptom onset in all patients irrespective of disease severity. Infectious viral shedding was noted only during the early acute phase of disease i.e., in the first week after symptom onset (innate immune response). All patients displayed cellular and humoral adaptive responses. Indeed, T-cell immune response was noted in all patients. However, the frequency of activated CD8 T cells was lower in patients with mild symptoms. Overall, the study supported most recommendations, which advise isolation for a minimum of 10 days, even in patients with mild symptoms.

A seroprevalence study was conducted within selected locations in Ghana to estimate the level of exposure of SARS-CoV-2 in individuals across a wide socioeconomic range. Using a strip-in-cassette lateral flow type RDT which separately detects IgM and IgG antibodies against the nucleocapsid protein of the virus, 252/1,305 persons tested positive for either of the antibodies or both (34). Exposure rates in August 2020 revealed a significantly higher level amongst persons of lower income, lower educational level and those engaged in informal employment. Between October and December 2020 there was no apparent increase in exposure rates indicating either a reduction in transmission intensity or loss of circulating antibody responses. In a more recent survey conducted in February 2021 during Ghana's second wave of infection, rates of exposure in persons of higher income almost doubled, leading to the loss in exposure stratification according to socioeconomic status, albeit most of the cases remaining asymptomatic.

Cheng et al. (24) reported a higher risk of infection at the onset of infection with the risk reducing at later stages of the disease. This observation was confirmed with empirical evidence of increased viral loads measured in throat swabs concomitant with symptoms which reduced to lower viral loads toward day 21 (26). Other (27) studies report that careful follow up among close contacts with asymptomatic COVID-19 infections did not differ whether the infected person had symptoms or not. Oma et al. (35), found that nasal shedding preceded fecal shedding and calves shedding BCoV RNA after 21 days of infection did not infect sentinel animals supporting the fact that prolonged shedding of BCoV RNA does not necessarily indicate transmission potential.

According to Kissler et al. (36), the transmission dynamics of SARS-CoV-2 is influenced by seasonal variation in transmission, the longevity of immunity, and the degree of “protection” provided as a result of exposure to other coronaviruses, as well as the frequency and timelines of control measures. Walsh et al. (37) summarized major findings from 113 studies on the evolution of SARS-CoV-2 viral load with time as well as the time frame during which the patient remains infectious. From evidence on the detection pattern and viral load of SARS-CoV-2 over the course of an infection (including any asymptomatic or pre-symptomatic phase), and the duration of infectivity, it was found that maximum viral RNA could be detected in upper respiratory tract samples few days after the onset of symptoms or during symptom onset. Around 2 weeks after symptom onset, no viral RNA could not be detected at all. Sputum samples showed higher viral loads, and persisted for longer time. Prolonged viral RNA detection in fecal specimens was noted. However, no study has been found whereby the duration of infectivity was definitively measured. Indeed, COVID-19 patients cannot transmit live virus during the entire period of viral RNA detection since the presence of viral ribonucleic acid may not necessarily indicate transmissible live virus. SARS-CoV-2 viral RNA load from respiratory tract samples displayed a rather consistent trend over the course of COVID-19 infection.

Zuo et al. (38) assessed the variation in SARS-CoV-2 transcriptional activity and tried to correlate the latter with changes in fecal microbiome. SARS-CoV-2 viral RNA samples were found in stool samples of 46.7% of the COVID-19 positive patients. Forty two percent of these patients showed active viral infection signature for a period of up to 6 days after clearance of SARS-CoV-2 from respiratory samples. The viral metagenome profile of all patients showed higher coverage (*p* = 0.0261) and density (*p* = 0.0094) of the 3′ end of SARS-CoV-2 genome compared to the 5′ end even if the patients did not display GI symptoms. Moreover, the gut microbiota of COVID-19 patients with GI infection showed increased amounts of opportunistic pathogens, as well as reduced salutary bacterial content. This important study showed the persistence of transcriptional activity of viral infection and replication in the gut even after clearance of SARS-CoV-2 from respiratory samples. Furthermore, the authors deciphered for the first time, the signature of active gut viral infection in COVID-19 patients even in the absence of GI symptoms, suggesting “quiescent” GI infection of SARS-CoV-2 following recovery from COVID-19.

### Transmission Potential and Shedding of SARS-CoV-2 in Convalescing Patients

Several studies have been conducted regarding viral shedding in nasopharyngeal swabs, tears, urine and excreta of severe, mild, and asymptomatic COVID-19 cases. Nonetheless, there are still knowledge gaps regarding the viral shedding dynamics during the convalescent phase in COVID-19 patients with mild and severe symptoms. Recent studies suggest that asymptomatic COVID-19 carriers may be as contagious as symptomatic (mild and severe) patients, and therefore may play an important role in transmission through viral shedding (39–41). However, in all these studies, viral RNA was detected via PCR and not via viral culture as a result of which it is difficult to conclude on whether the detection of viral RNA by RT-PCR was related to viable virus or shedding of remnant non-viable genetic material. Indeed, detection of viral subgenomic RNA correlated poorly with shedding of infectious virus. These RNAs are produced only in actively infected cells and are not packaged into virions. Subgenomic RNAs were still detected when virus cultures turned negative. This could indicate that active replication continues in severely-ill symptomatic COVID-19 patients after seroconversion and after shedding of infectious virus has stopped. Possibly, infectious virions are produced but are directly neutralized by antibodies in the respiratory tract. On the other hand, the half-life of viral subgenomic RNAs is not known in COVID-19 and these RNAs may still be detected once replication has stopped. In addition, asymptomatic patients recruited in the studies may not be a good representation due to false-positives. Despite limitations presented by these studies, the potential of SARS-CoV-2 viral transmission via asymptomatic patients cannot be underestimated. Therefore, an understanding of the viral burden and transmission potential of patients whose symptoms have resolved through larger epidemiologic investigations will be useful to design appropriate strategies for epidemiological management of the disease.

COVID-19 patients displaying mild symptoms were assessed for any unusual signs and their symptoms were correlated with the viral shedding period. RT-PCR was performed every 2–7 days, with the duration of viral shedding ranging from the day of first diagnosis to the day before the first negative test. On average, viral shedding lasted for 24.5 days. According to this study, it was evident that all patients presenting with chest pain and sputum shed the virus significantly longer than patients without these symptoms. Zhou et al. (42) studied the risk factors of in treatment facility's death rate for patients infected with SARS-CoV-2, the duration of clinical symptoms, viral shedding, and the associated differing laboratory findings in the course of hospitalization using a retrospective multi-center cohort approach. A total of 191 study subjects were successfully followed up to the end of the study. Out of these, 54 patients died whereas 137 recovered and were discharged. The patients' ages ranged between 18 and 87 years, and about half of them had comorbidities. The average duration of illness to discharge was 22 days (IQR 18–25), whereas the average time to mortality was 18.5 days (IQR 15–22). For survivors, viral RNA was detectable through a duration of 20 days from the onset of illness. However, in non-survivors, viral RNA was detectable until death. The longest and shortest observed duration of viral shedding among survivors was 37 and 8 days, respectively. For patients who received anti-viral therapy, the number of days of viral shedding differed slightly according disease status. For those who had severe illness viral shedding persisted for 19 days (17–22) whereas for patients with critical illness status their viral shedding persisted for 24 days (22–29, 43). Shortcomings of this study included (1) the lack of effective antivirals, inadequate adherence to standard supportive therapy, and high-dose corticosteroid use, (2) limited frequency of respiratory specimen collection, (3) lack of quantitative viral RNA detection, (4) limited sample size, and (5) late transfer of patients to hospitals. Nevertheless, the authors argue that this study is crucial to consider in patient management during isolation and the guidance concerning the length of antiviral treatment given that effective antiviral treatment may improve clinical outcomes in COVID-19.

Yan et al. (44) assessed the factors associated with long-term viral shedding and effect of lopinavir (LPV) or ritonavir (r) treatment on hospitalized COVID-19 patients in China. A comparison of the clinical features and SARS-CoV-2 viral shedding was conducted between patients treated with and without LPV/r. SARS-CoV-2 RNA could be detected for a median duration of 23 days from symptom onset. Older age and the lack of LPV/r treatment were identified as independent risk factors for long and persistent viral shedding. Indeed, patients treated with LPV/r within the first 10 days of symptom onset showed significantly shorter viral shedding duration compared to those who did not receive LPV/r treatment (median 19 vs. 28.5 days). The work of Yan et al. (44) concluded that administration of LPV/r at an early stage of SARS-CoV-2 infection could reduce the duration of viral shedding.

## Extra-Long Duration OF Viral Shedding/Viral RNA Detection

Li et al. (45) observed persistent shedding of SARS-CoV-2 60 days from onset of typical symptoms in a 71-year old Chinese woman with prolonged shedding 36 days after recovery. This observation suggested that asymptomatic, mildly symptomatic and recently recovered patients may require prolonged isolation. However, since the report by Li et al. (45) is based on a single patient, further investigations based on larger cohorts will be required to support this conclusion. In another study (46), characterization and subsequent analysis of epidemiological, clinical, laboratory, etiologic detection and radiological features of COVID-19 demonstrated that for the moderate form of COVID-19, individuals could shed viral particles for up to 25 days. Limitations of this study consisted of the following: (1) lack the data during the first week of infection as most patients were transferred from other hospitals, (2) limited frequency of sample collection and the lack of quantitative viral RNA detection, and (3) lack the data for stool samples. Man et al. (47) reported viral shedding prolongation in a kidney transplant patient with COVID-19 pneumonia on days 57 and 63 which surprisingly turned out positive after relief of symptoms. This study also suggested that recovered patients may shed viruses for a median duration of 20 days. The longest reported duration of viral SARS-CoV-2 RNA shedding from upper respiratory tract was 83 days (45). The median duration of viral shedding from the time of onset of symptoms in upper respiratory tract samples was 19 days (IQR: 14–25 days) compared to 34 days (IQR: 24–40 days) in lower respiratory tract samples (48). SARS-CoV-2 RNA seemed to persist longer in lower respiratory tract samples compared to upper respiratory tract samples (6, 49).

Liu et al. (11) successfully related the viral load to the viral shedding period whereby higher viral load in severe COVID-19 cases led to longer viral shedding period. Indeed, upper respiratory tract specimens tested negatively 10 days after manifestation of symptoms in 90% of mild cases in contrast to severe cases where tests remained positive for a longer time (11). In addition, the median duration of viral detection in pre-symptomatic patients was 12 days compared to 6 days in asymptomatic ones (50). Moreover, viral RNA could be detected for longer time in males (vs. females) and in older patients possibly due to differences in immune status/ hormone levels and increased ACE-2 concentrations (6).

Children with mild COVID-19 were clinically assessed at Wuhan Children's Hospital, Wuhan, China and their laboratory test results were also investigated and correlated with viral shedding (51). On average, 6-year old children shed SARS-CoV-2 virus for 15 days as calculated from the onset of COVID-19 to hospital discharge. This time frame was smaller in patients without symptoms compared to those presenting symptoms (11 vs. 17 days). Age <6 years [odds ratio (OR) 8.94], hypersensitive C-reactive protein level >3.0 mg/L (OR 4.89) and pneumonia (OR 8.45) correlated with a higher probability of symptomatic infection. Children displaying COVID-19 symptoms, fever as a result of pneumonia and lymphocyte concentration <2.0 × 10^9^/L shed SARS-CoV-2 virus for longer time. Lu et al. (51) suggested that close follow-up of symptoms could be associated with viral shedding in COVID-19 infected children.

Park et al. (52) determined the persistence of SARS-CoV-2 virus in patients displaying symptoms and also assessed the duration of RNA detection in nasopharyngeal/oropharyngeal swabs or sputum or saliva in 6 patients using real-time reverse transcriptase polymerase chain reaction. SARS-CoV-2 virus could be detected in patients at a median time of 34 days (11, 22–66) following their hospitalization. Furthermore, the virus could still be detected in patients even after symptom resolution for a median duration of 26 days (9–48). In one of the patients, viral RNA could persistently be detected until 67 days of hospitalization i.e., 30 days following resolution of symptom. This is the longest time period of SARS-CoV-2 detection and reiterates the importance of long-term patient follow up even after symptom resolution.

Li et al. (53) assessed the dynamics of viral RNA shedding at various stages in COVID-19 infected patients. Pre-symptomatic, asymptomatic and mildly symptomatic patients showed median SARS-CoV-2 shedding of 11.5, 28, and 31 days, respectively. 38.9% of patients displayed persistent shedding after hospital discharge. Antibodies to SARS-CoV-2 and viral RNA could be simultaneously detected in 27.8% of patients during the convalescent phase. Long-term RNA shedding was noted in patients presenting mild symptoms as well as in asymptomatic ones. The authors suggested that specific antibody production may not imply SARS-CoV-2 clearance after hospital discharge and this is an important point to be considered when deciding on strategies to better control SARS-CoV-2 infection.

Lee et al. (40) reported that in a retrospective cohort of 201 symptomatic COVID-19 patients, the median RNA shedding time was 14 days (IQR 9–18) and 38.3% of patients showed intermittent viral shedding. The duration of shedding displayed an inverse correlation with plasma levels of T-cell cytokines IL-1β and IL-17A at the initial phase of infection, and the levels of pro-inflammatory cytokines were lower in patients during intermittent shedding. The less active T-cell responses at the start of COVID-19 infection could be correlated with prolonged viral RNA shedding and thus early immune responses help to manage viral load and to prevent viral RNA shedding.

In a recent study conducted on 20 immunocompromised COVID-19 patients, it was found that viral shedding occurred for up to 78 days after symptom onset (54). Interestingly, follow-up specimens taken from 5 patients showed the presence of viable virus post 8, 17, 25, 26, and 61 days after the onset of symptoms. Three of these patients underwent allogeneic hematopoietic stem-cell transplants (2 patients) or CAR T-cell therapy (1 patient) within the previous 6 months. Overall, this important study highlights the fact that immunosuppressed patients may continue shedding viable SARS-CoV-2 for at least 2 months and thus the current WHO discharge guidelines may not be convenient for these patients.

Kim et al. showed that the time from disease onset to viral clearance in culture with the time to clearance in real-time RT-PCR tests was not in agreement with each other (55). In fact, following viral cultures and real-time RT-PCR, the median time for viral clearance was found to be 7 and 34 days, respectively. Positive viral cultures were obtained only in samples with a cycle-threshold value of 28.4 or less. They noted that as the time from symptom onset increased, the probability of culture positivity decreased. However, conclusions from this study need to be cross-checked in larger and more diverse groups of patients due to the small sample size, inconsistency in timing of sampling, and relatively mild illness of the enrolled patients.

## Viral Viability and Infectivity in Different Scenarios

Even though evidence showed that SARS-CoV-2 viral shedding occurs both in patients with and without symptoms, the correlation between transmissibility/infectivity and detectable viral RNA remains unclear (56). This is due to the fact that a positive RT-PCR result does not necessarily imply the possibility of viral transmission as this test cannot differentiate between infective and inactive virus and a higher amount of viral RNA does not necessarily imply greater infectivity. Another aspect to be considered in this matter is seroconversion. It is still not clearly understood how seroconversion is related to infectiousness. Wölfel et al. (8) and Liu et al. (11) noted the persistence of viral shedding after seroconversion and reported on the successful culture of SARS-CoV-2 virus after the detection of antibodies.

Only few studies have correlated RT-PCR test results with viral cultures and infectivity. Arons et al. (22) could successfully isolate viable viruses from specimens collected 6 days before to 9 days after the appearance of first typical symptoms. These authors reported that 67.2% of specimens collected from the upper respiratory tract resulted in positive viral cultures. Viral load values (Ct) as low as 34.3 gave positive culture growth. In addition, positive viral cultures could be obtained from asymptomatic, pre-symptomatic and symptomatic patients (22). Detection of infectious isolates depended on the sample site. Indeed, SARS-CoV-2 virus could be readily isolated from throat and lung derived samples in contrast to stool samples despite high viral loads (8). Despite detection of SARS-CoV-2 RNA by PCR 20 days after onset of symptoms, isolation of the latter was not possible even though viral loads were high (57). Moreover, viral culture positivity rate decreased progressively with increasing Ct values until no culture could be obtained with Ct values higher than 34 targeting the E gene.

The extent and length of infectious virus replication should be considered when evaluating the possibility of viral transmission which will in turn guide decisions regarding patient discharge and isolation. Qualitative or quantitative viral RNA tests have been used extensively as a potential marker for infectious coronavirus since RNA detection is more sensitive than virus isolation. But this can be problematic because, shedding viral RNA may not necessarily be synonymous to shedding whole viruses. Even though assessing the potential infectivity is a labor-intensive process, it is not possible to estimate the average time frame of viral RNA shedding by investigating the presence of SARS-CoV-2 viral RNA in patient samples as the presence of nucleic acid alone does not necessarily imply infectivity (58). Several studies have shown that viral nucleic matter can still be quantified in diseases such as SARS-CoV-1, MERS, influenza virus, Ebola virus, Zika virus, etc. even though the viable virus cannot be detected (59–63) and in fact, measles viral RNA can still be detected 6–8 weeks after the clearance of infectious virus. It is believed that the immune system can neutralize viruses by solubilizing or disintegrating the viral envelope or aggregating virus particles; thus, preventing viral transmission without eliminating nucleic acid, which eventually degrades (59–63). This is because the detection methods of nucleic acids, i.e., the PCR method cannot be used to differentiate between infectious virus and non-infectious RNA. Thus, PCR findings should be interpreted with care and caution must be exercised especially when taking decisions on isolation policies, as infectivity data are critical to demonstrate these specific aspects.

The critical question in the ensuing discussion so far in this review, is how long a person continues to shed infectious virus? In order to answer this question, it is important to note that the determinant of infectious virus is not the detection of sub-genomic RNA which is widely used in testing centers, but *in vitro* infectiousness on cell lines by replication competent virus is regarded as a more informative surrogate of viral transmission (64–66). A number of studies have demonstrated that infectious virus could not be detected in respiratory tract samples obtained more than 8 days after onset of symptoms despite continued detection of high levels of viral RNA (8, 67). This is the evidence that gives credence to the WHO patient discharge protocol. However, new evidence has emerged, suggesting that in patients with severe to critical cases shedding infectious virus for more than 10 days are common, due to their higher viral loads. Liu et al. (11, 49) reported a single case of mild COVID-19 in which infectious viruses were reported for 18 days after the onset of symptoms.

Van Kampen et al. (30) studied 690 respiratory samples from 129 severe to critical patients using cell culture assay, viral RNA load with RT-qPCR as well as measurement of neutralizing antibody titres. Infectious SARS-CoV-2 was isolated from 62 respiratory tract samples of 23 patients in which detection of infectious virus was common after 8 days or more since onset of symptoms. For a single patient, infectious virus was detected up to 20 days after onset of symptoms. Shedding of infectious virus up to 18 days after onset of symptoms has been reported for a single case of mild COVID-19 (11, 49). Higher viral loads have been reported for severe COVID-19 cases compared to mild cases, which may in part explain the longer duration of shedding found in this study (6, 11, 26, 68). Kim et al. (55) studied 20 patients, of which 11 had severe COVID-19. A total of 78 samples were collected from the 20 patients; 57 samples at various time periods. Viral RNA was detected for up to 78 days after the onset of symptoms. However, viable virus was detected in 10 of 14 nasopharyngeal samples (71%). Out of these, samples from 5 patients replicated viruses in culture for 8, 17, 25, 26, and 61 days after the onset of symptoms. Three patients who shed live viruses for more than 20 days had profound immunosuppression and had no neutralizing antibodies (were seronegative) and were actually receiving hematopoietic cells as therapy.

Economic challenges and prolonged turn-around times of rRT-PCR-based testing makes it inadequate for post-discharge screening of potentially infectious individuals. For these reasons, antigen-based screening for SARS-CoV-2 is being considered as a complement to rRT-PCR-based testing (69). Studies have demonstrated sensitivity of SARS-CoV-2 Ag-RDTs between 22.9 and 93.9% compared to rRT-PCR (70–78). However, antigen-based tests have been found to be comparable with culture-based tests (79, 80). Separate reports by Kohmer et al. (81) and Pekosz et al. (82) have recently shown that large-scale SARS-CoV-2 Ag-RDT-based point-of-care testing can be considered for detecting potentially contagious virus and reduce SARS-CoV-2 transmission. In light of these reports, we believe that SARS-CoV-2 antigen testing, if deployed for post-discharge screening of COVID-19 patients particularly in resource-limited settings, can facilitate cost-effective and timely detection of individuals harboring infectious viruses as well as provide risk determination of the present WHO discharge guidelines.

## Influence of Environmental Factors on Viral Transmission and Viability

The most common mode of human to human transmission is via body fluid droplets, infected hands and surfaces (83). The SARS-CoV-2 virus was still infectious after 3 h when dispersed in aerosols while it showed higher viability of 4, 24 and 72 h in the form of droplets on copper, cardboard, plastic and stainless steel, respectively (84). In contrast, the viability of the virus in aerosols was only 3 h (84). In addition, SARS-CoV-2 virus was found to be stable for 2 and 7 days on rough surfaces (fabric and wood) and smooth surfaces (steel and plastic), respectively (85).

Environmental conditions including temperature, humidity, wind speed, water, sewage, air, insects, and surface of objects influence the viral transmission process. The prevalence of COVID-19 with maximum air humidity and wind speed was found to be negligible (86) while prevalence decreased with increasing temperature, with unit increase in the minimum surrounding air temperature resulting in a 0.86% decrease in the cumulative number of cases (16). In another study, the daily mortality of COVID-19 was negatively associated with absolute humidity (87). Following investigation in Chinese and USA cities, a decrease in reproductive number (R) of SARS-CoV-2 was noted with increased temperature and humidity (88).

Few studies have tried to investigate the correlation between air pollution and COVID-19 cases. Wu et al. (89) observed that a slight increase (1 μg/m3) in the concentration of particulate matter increased the number of COVID-19 fatalities. In line with this, in a cross-sectional nationwide study, Liang et al. (90) found showed that COVID-19 participants who were exposed to NO_2_ for a long time had higher death probability (90). Furthermore, up to 78% of 4,443 COVID-19 fatality cases considered by Ogen (91) corresponded to main NO_2_ hotspots in Europe. Similarly, Zhu et al. (92) found that short term exposure to air pollutants such as PM2.5, PM10, CO, NO_2_, and O3 led to increased risk of COVID-19 infection. In summary, most studies indicate that patients developed more severe forms of COVID-19 when they were exposed to air pollutants for long time and also took longer to recover and had increased mortality rates.

CDC has not identified food, food packages, and food handlers as a major mode of viral transmission. However, washing and disinfection of surfaces is highly recommended based on evidence suggesting the persistence of the virus for hours/days (93). Most importantly, complete personal hygiene such as covering the nose and mouth when sneezing/ coughing is recommended for food handlers and those involved in food preparation. The detection of SARS-CoV-2 RNA in sewage, even though at low concentration shows that the virus can survive in wastewater (94).

In addition to environmental factors, human population density and movement, social interactions, climate change also impacts on viral transmission.

## Recommendations

The WHO played an important role in issuing guidelines and measures to control the worldwide COVID-19 pandemic. In line with this, the latter provided a first set of recommendations for the discharge of COVID-19 patients. Due to challenging conditions and poor infrastructure in many countries, these recommendations were reviewed. Following a critical assessment of follow-up studies involving convalescing COVID-19 patients, we posit that there is no strong evidence which counters the current WHO recommendations. However, we propose the use of a test-based discharge strategy in immunocompromised individuals. RT-PCR tests are less reliable. Indeed, false-negative have been reported due to poor quality specimen collection, testing too early in the incubation period, and other processing errors. LRT specimens were shown to be more sensitive compared to URT ones. Viral loads could be correlated with disease severity with higher viral loads noted for severe forms of COVID-19. However, viral load and viral shedding cannot be directly correlated with infectivity because RT-PCR based assays do not distinguish between degraded viral genetic remains or viable virus. In patients with critical COVID-19, infectious virus may be shed for longer periods in contrast to the WHO guidelines. It has been observed that in the presence of serum neutralizing antibodies, infectious virus shedding drops to undetectable levels below a viral RNA load threshold. This suggests that quantitative viral RNA load and immunological assays are better alternatives in test-based approaches when deciding to relax infection prevention and control precautions. Van Kampen et al. (30) observed that once the serum neutralizing antibody titer of at least 1:80 is achieved, infectious viruses could not be isolated from respiratory tract samples of patients. These pieces of evidence suggest that serological assays and quantitative viral loads must be used in test-based policies when deciding to ease control or prevention methods.

## Author Contributions

All authors conceived the literature review concept, conducted a literature search, compiled and interpreted all data, and wrote the entire manuscript. All authors have read and approved the entire manuscript.

## Conflict of Interest

The authors declare that the research was conducted in the absence of any commercial or financial relationships that could be construed as a potential conflict of interest.
